# The associated factors of smoking cessation intention among husbands in gestational households: A census-based cross-sectional survey

**DOI:** 10.18332/tid/213719

**Published:** 2026-01-09

**Authors:** Xiaokai Wang, Fangyuan Yu, De Chen, Xuelian Chen, Qingwei Li, Jiani Ding, Yifang Chen

**Affiliations:** 1 Department of Shanghai Jinshan District Disease Prevention Center, Shanghai, China; 2 Shanghai Municipal Center for Health Promotion, Shanghai, China; 3 Department of Shanghai Jinshan District Maternity and Child Care Centers, Shanghai, China

**Keywords:** smoking cessation intention, gestational households, secondhand smoke

## Abstract

**INTRODUCTION:**

Secondhand smoke (SHS) exposure poses health risks to pregnant women, with spousal smoking identified as the primary source of SHS exposure among Chinese pregnant women. This study examines smoking cessation intention and its associated factors among smoking husbands during their wives' pregnancy.

**METHODS:**

This was a cross-sectional study in Shanghai from April 2021 to December 2024, enrolling all registered pregnant women and their cohabiting smoking husbands in Jinshan District, Shanghai (n=1754 couples). Separate structured questionnaires were administered to collect demographic data and smoking-related behaviors. Double data entry was performed using EpiData 3.1, with SPSS 26.0 employed for statistical analyses.

**RESULTS:**

Among the participants, 47.4% of the smoking husbands expressed a willingness to quit smoking. The results of the univariate analysis indicate that a husband's willingness to quit smoking is associated with several factors, including his age, health status, and the surrounding environment (p<0.05). Multivariable logistic regression analysis revealed that husbands' willingness to quit smoking was significantly associated with several factors. Specifically, compared to husbands younger than 25 years old, those older than 35 years were less willing to quit smoking (adjusted odds ratio, AOR=0.52; 95% CI: 0.38–0.70). Additionally, husbands in poor health were less likely to quit than those in good health (AOR=0.65; 95% CI: 0.50–0.93). Furthermore, husbands with higher levels of education were less likely to quit than those with lower levels (AOR=0.62; 95% CI: 0.46–0.82). When comparing smoking habits, husbands who smoked ≥11 cigarettes per day were less willing to quit than those who smoked ≤5 cigarettes daily (AOR=0.56; 95% CI: 0.42–0.75).

**CONCLUSIONS:**

Smoking cessation intentions among husbands are influenced by multidimensional interactions of individual characteristics (age, health status), smoking behavior patterns (intensity, duration), and family and social environments (smoking bans, peer influence). Future research should elucidate the mechanisms underlying the interactions among these variables.

## INTRODUCTION

Smoking stands as a major risk factor for preventable deaths globally, closely associated with the occurrence and progression of significant diseases such as cardiovascular diseases and malignancies, posing a persistent threat to public health systems^1,2^. Furthermore, the health hazards of smoking behavior are not confined to active smokers; the issue of secondhand smoke (SHS) exposure, which is associated with increased risks of ischemic heart disease, stroke, type 2 diabetes, and lung cancer, among others, is equally worthy of attention^3^. Epidemiological data reveal that the rate of secondhand smoke exposure among non-smokers in China is as high as 68.1%^4,5^. Among pregnant women, the issue of secondhand smoke exposure is particularly prominent. As core members of their living environment, their spouses’ smoking habits often become the source of secondhand smoke exposure for pregnant women^6^. Exposure to secondhand smoke during pregnancy can significantly increase the risks of preterm birth, low birth weight, and congenital malformations through mechanisms such as nicotine-mediated placental vasoconstriction and carbon monoxide-induced fetal hypoxia^7,8^. Given the potential severe threats posed by secondhand smoke to the health of pregnant women and fetuses, it is particularly crucial for husbands to successfully quit smoking during pregnancy. However, a study revealed that among 466 expectant fathers who smoke in China, 67.5% reported having no intention to quit smoking within the next 30 days following their partner’s pregnancy^9^, a figure far below the actual social demand and widespread expectations. Therefore, exploring effective ways to enhance the smoking cessation intention of husbands who smoke during pregnancy has become an important issue that warrants further research.

Behavior-change theories indicate that the perinatal period serves as a ‘critical window’ for family health concerns. During this stage, family members’ concern for the health of pregnant women and fetuses often prompts them to actively listen to and accept advice on smoking cessation^10,11^. In view of this, this study focuses on the group of smoking husbands in pregnant families. This study aims to understand their smoking cessation intentions and the multifaceted factors. The findings are expected to contribute to the scientific knowledge base for developing health promotion strategies for pregnant families. This has significance for reducing secondhand smoke exposure among pregnant women and improving birth outcomes. Furthermore, the evidence generated can inform the public health strategies in developing regions.

## METHODS

### Participants

This population-based, cross-sectional study was conducted in Jinshan District, Shanghai, from April 2021 to December 2024. The study specifically targeted pregnant women who registered for prenatal care at community health service centers and hospitals in Jinshan District, along with their cohabiting husbands who were current smokers. To ensure the accuracy and consistency of the study subjects, the following inclusion criteria were applied: 1) permanent residents who have lived in the area for more than six months (including local registered residents and long-term non-local residents); 2) women currently in their pregnancy; 3) husbands of the pregnant women are smokers; and 4) both the pregnant women and their husbands voluntarily participated in this study and were capable of completing the questionnaires. This study has been approved by the Ethics Committee of the Jinshan District Center for Disease Control and Prevention, Shanghai (Ethics Approval Number: JCZXERC-202307). All participants signed informed consent forms when completing the questionnaires, and all data used for analysis were anonymized.

### Questionnaire

This study utilized a self-designed questionnaire, which was partially adapted from the International Tobacco Control Policy Evaluation Project, ITC^12^. Separate questionnaires were administered to pregnant women and their husbands. The pregnant women’s version covered demographic information (age, gestational week, parity, education level, occupation), secondhand smoke exposure (frequency, locations), household atmosphere (smoking status of cohabiting members, indoor smoking frequency), as well as attitudes toward smokers and awareness of SHS health effects. The husbands’ questionnaire collected corresponding demographic data, smoking history (years smoking, daily cigarette consumption, smoking indoors or near the pregnant woman), smoking cessation history and willingness to quit (previous attempts, quit duration, methods used, future plans), plus attitudes toward smoking and understanding of SHS hazards.

Variables were clearly defined and categorized to support consistent analysis. Continuous variables such as age were grouped according to clinical and distributional relevance, e.g. husbands’ age was categorized as <26, 26–30, and >30 years; daily cigarette consumption was classified as <6, 6–10, and >10 cigarettes; and smoking duration was categorized as <6, 6–10, and >10 years. Categorical variables included health status (good, poor), presence of chronic diseases (yes, no), household smoking policy (complete ban/partial or no restrictions), and family members’ attitudes toward smoking (opposed, tolerant). Education level was grouped as high school or lower versus college or higher, and nicotine dependence was assessed using validated scales and classified as mild, moderate, or severe. Nicotine dependence was assessed using the Fagerström test for nicotine dependence (FTND) scale, with scores categorized into three levels: mild (0–3 points), moderate (4–6 points), and severe (7–10 points)^13^.

### Data collection

This study is grounded in the Chinese Maternal Health Service Standards, which stipulate that ‘pregnant women must visit designated medical institutions within 12 weeks of pregnancy to establish a maternal health record and complete prenatal check-up registration’^14^. During the registration process, trained investigators screened eligible pregnant women and their spouses according to pre-established inclusion criteria. If the spouse accompanied the pregnant woman to the visit, the investigator conducted face-to-face interviews with both the pregnant woman and her spouse on-site. Conversely, if the spouse did not accompany the pregnant woman, the investigator provided her with a unique QR code, which she would then share with her spouse to access the online questionnaire system and complete the questionnaire within the study’s stipulated time frame. The study population comprised all pregnant women who registered for prenatal care in Jinshan District, Shanghai, between April 2021 and December 2024. Eligible participants were subsequently identified and enrolled based on the predefined inclusion criteria.

### Quality control

All investigators underwent standardized training. During on-site investigations, quality control measures were implemented, ensuring the completeness of the questionnaires and addressing any missing or incomplete items on the spot. For participants unable to complete the questionnaire on-site, information was supplemented through telephone follow-ups.

### Statistical analysis

Epidata 3.1 was utilized for double data entry, while SPSS 26.0 was employed for statistical analysis. Descriptive statistics were computed to summarize the general attributes of the survey participants, including means and standard deviations (SD) for continuous variables, and frequencies (n) and percentages (%) for categorical variables. Chi-squared analysis was applied to evaluate the husband’s intention to quit smoking. Additionally, multivariate logistic regression analysis was performed on the statistically significant findings from the univariate analysis, with a significance level set at α=0.05.

Following the univariate analysis, 20 factors exhibiting statistically significant differences in smoking cessation intention were included in the logistic regression analysis. These factors were: husband’s age, health status, chronic illness, education level, pregnant woman’s age, number of pregnancies, pregnant woman’s smoking status, daily smoking amount, years smoked, whether used e-cigarettes, number of quits, nicotine dependence level. And awareness that: smoking causes stroke, smoking causes heart disease, smoking causes impotence, secondhand smoke causes heart disease, secondhand smoke causes adverse pregnancy. Finally, whether smoking is allowed at home, family members’ attitudes towards smoking, and number of smokers in husband’s environment. The logistic regression analysis was conducted with the presence of smoking cessation intention as the dependent variable [yes=1 (reference), no=0], allowing for observation of changes in the dependent variable as each independent variable varies. Additionally, each variable was assigned a corresponding value.

## RESULTS

From April 2021 to December 2024, a total of 1754 pregnant families participated in this study, comprising 1754 pregnant women and their smoking husbands. The average age of the pregnant women was 29.0 ± 5.2 years, while the average age of their husbands was 32.5 ± 5.4 years. Among the surveyed pregnant women, 24.6% were local residents of Shanghai, whereas 75.4% were non-local residents. The proportion of pregnant women with a high school education level or lower was 54.7%, compared to 81.6% among their husbands. Additionally, 39.9% of the pregnant women were primiparas. Detailed statistical results are presented in Supplementary file Table 1.

The results of this study indicate that 47.4% of the husbands expressed a willingness to quit smoking. Further analysis revealed that 64.1% of husbands aged ≤26 years were willing to quit, compared to 44.6% of those aged ≥30 (p<0.05). Among husbands in good health, 50.7% were willing to quit, whereas this proportion decreased to 42.5% among those in poor health (p<0.05). Additionally, 45.8% of husbands with chronic diseases were willing to quit, while this proportion increased to 61.6% among those without chronic diseases (p<0.05). Husbands with a lower level of education exhibited a higher willingness to quit (48.8%) (Table 1).

**Table 1:** Univariate analysis of smoking cessation intention and demographic characteristics of husbands, Shanghai, China, 2021–2024 (N=1754)

*Characteristics*	*Willing to quit smoking n (%)*	*No desire to quit smoking n (%)*	*χ^2^*	*p*
**Age** (years)			17.821	<0.001
<26	75 (64.1)	42 (35.9)		
26–30	266 (49.7)	269 (50.3)		
>30	491 (44.6)	611 (55.4)		
**Occupation**			5.204	0.391
Agricultural labor	35 (47.9)	38 (52.1)		
Civil servant	48 (54.5)	40 (45.5)		
Private enterprises	392 (47.6)	432 (52.4)		
Professional technicians	17 (54.8)	14 (45.2)		
Other	327 (45.6)	390 (54.4)		
Unemployed	13 (61.9)	8 (38.1)		
**Health status**			8.475	0.014
Good	470 (50.7)	457 (49.3)		
Average	325 (43.9)	415 (56.1)		
Poor	37 (42.5)	50 (57.5)		
**Chronic illness**			15.803	<0.001
Yes	723 (45.8)	854 (54.2)		
No	109 (61.6)	68 (38.4)		
**Education level**			5.618	0.018
High school and lower	698 (48.8)	733 (51.2)		
College and higher	134 (41.5)	189 (58.5)		

The outcome variable ‘willingness/intention to quit’ was defined based on the question: ‘Do you intend to quit smoking within the next 30 days?’ Responses were collected on a 5-point scale: 1=definitely yes, 2=probably yes, 3=undecided, 4=probably not, and 5=definitely not. For analysis, the variable was dichotomized into ‘willing’ (categories 1–2) and ‘not willing’ (categories 3–5).

The research findings indicate that among families experiencing their first pregnancy, 51.3% of husbands expressed a willingness to quit smoking. In contrast, this proportion decreased to 42.2% among husbands of women with more than two pregnancies (p<0.05). Furthermore, among pregnant women who actively smoke, 36.6% of their husbands indicated a willingness to quit smoking, compared to 50.4% among husbands of non-smoking pregnant women (p<0.05) (Table 2).

**Table 2:** Univariate analysis of husband’s smoking cessation intention and demographic characteristics of pregnant women, Shanghai, China, 2021–2024 (N=1754)

*Characteristics*	*Willing to quit smoking n (%)*	*No desire to quit smoking n (%)*	*χ^2^*	*p*
**Age** (years)			15.922	<0.001
<26	229 (54.3)	193 (45.7)		
26–30	287 (48.9)	300 (51.1)		
>30	316 (42.4)	429 (57.6)		
**Occupation**			1.452	0.919
Agricultural labor	21 (55.3)	17 (44.70)		
Civil servant	33 (47.1)	37 (52.9)		
Private enterprises	235 (46.3)	273 (53.7)		
Professional technicians	54 (46.2)	63 (53.8)		
Other	379 (47.7)	416 (52.3)		
Unemployed	110 (48.7)	116 (51.3)		
**Education level**			0.406	0.524
Lower than university	462 (48.1)	498 (51.9)		
University and postgraduate	370 (46.6)	424 (53.4)		
**Number of pregnancies**			10.213	0.006
1	359 (51.3)	341 (48.7)		
2	244 (47.7)	267 (52.3)		
>2	229 (42.2)	314 (57.8)		
**Smoking status**			6.989	0.030
Active smoking	15 (36.6)	26 (63.4)		
Passive smoking	388 (45.0)	474 (55.0)		

The results indicated a significant association between a husband’s smoking habits and his willingness to quit. Specifically, husbands who smoked <6 cigarettes per day exhibited a higher intention to quit smoking (61.0%), which was significantly greater than that of husbands who smoked >10 cigarettes per day (30.1%) (p<0.05). The proportion of husbands who had smoked for <6 years was 66.9%, notably higher than that of husbands who had smoked for >10 years (30.3%) (p<0.05). Additionally, husbands who used e-cigarettes demonstrated a greater intention to quit smoking, reaching 62.7%, compared to those who had never used e-cigarettes (45.1%) (p<0.05). The percentage of husbands who had never attempted to quit smoking was 37.8%. Among those husbands who had tried to quit smoking more than five times, 83.2% expressed a willingness to quit (p<0.05). Furthermore, nicotine dependence emerged as an important factor influencing the willingness to quit smoking. The study revealed that husbands with mild nicotine dependence had a smoking cessation intention of 56.1%, while those with severe nicotine dependence exhibited a lower intention of 33.0% (p<0.05) (Table 3).

**Table 3:** Univariate analysis of smoking cessation intention and smoking status of husbands, Shanghai, China, 2021–2024 (N=1754)

*Characteristics*	*Willing to quit smoking n (%)*	*No desire to quit smoking n (%)*	*χ^2^*	*p*
**Daily cigarette consumption**			117.272	<0.001
<6	487 (61.0)	311 (39.0)		
6–10	232 (39.9)	349 (60.1)		
>10	113 (30.1)	262 (69.9)		
**Years smoked**			149.314	<0.001
<6	401 (66.9)	198 (33.1)		
6–10	321 (40.5)	471 (59.5)		
>10	110 (30.3)	253 (69.7)		
**E-cigarette use**			24.984	<0.001
Yes	146 (62.7)	87 (37.3)		
No	686 (45.1)	835 (54.9)		
**Number of quit attempts**			102.343	<0.001
0	240 (37.8)	395 (62.2)		
1	188 (44.4)	235 (55.6)		
2–5	285 (51.5)	268 (48.5)		
>5	119 (83.2)	24 (16.8)		
Nicotine dependence*			69.711	<0.001
Mild	553 (56.1)	433 (43.9)		
Moderate	182 (38.4)	292 (61.6)		
Severe	97 (33.0)	197 (67.0)		

* Nicotine dependence levels were defined as: mild (FTND score 0–3), moderate (FTND score 4–6), and severe (FTND score 7–10). FTND: Fagerström test for nicotine dependence.

The results of the study indicate that non-pulmonary health risks associated with smoking and exposure to secondhand smoke include cardiovascular disease (including heart disease and stroke), sexual dysfunction (impotence), and adverse pregnancy outcomes. The results indicate that higher awareness of these health risks among husbands was associated with a greater intention to quit smoking. Specifically, higher awareness corresponds to a higher intention to quit. Conversely, analysis revealed no significant association between husbands’ awareness of the potential for smoking and secondhand smoke to cause lung disease and their smoking cessation intention (Table 4).

**Table 4:** Univariate analysis of smoking cessation intention and tobacco-related knowledge, Shanghai, China, 2021–2024 (N=1754)

*Items*	*Willing to quit smoking n (%)*	*No desire to quit smoking n (%)*	*χ^2^*	*p*
**Does smoking cause disease?**			1.212	0.271
Yes	684 (48.1)	739 (51.9)		
No	148 (44.7)	183 (55.3)		
**Does smoking cause stroke?**			8.634	0.003
Yes	432 (51.1)	414 (48.9)		
No	400 (44.1)	508 (55.9)		
**Does smoking cause heart disease?**			12.733	<0.001
Yes	459 (51.6)	430 (48.4)		
No	373 (43.1)	492 (56.9)		
**Does smoking cause lung cancer?**			0.005	0.943
Yes	695 (47.5)	769 (52.5)		
No	137 (47.2)	153 (52.8)		
**Does smoking cause impotence?**			10.375	0.001
Yes	453 (51.2)	431 (48.8)		
No	379 (43.6)	491 (56.4)		
**Does secondhand smoke cause diseases?**			3.213	0.073
Yes	686 (48.5)	729 (51.5)		
No	146 (43.1)	193 (56.9)		
**Does secondhand smoke cause heart disease?**			13.480	<0.001
Yes	495 (51.4)	468 (48.6)		
No	337 (42.6)	454 (57.4)		
**Does secondhand smoke cause pulmonary disease?**			0.156	0.693
Yes	633 (47.7)	694 (52.3)		
No	199 (46.6)	228 (53.4)		
**Does secondhand smoke cause lung cancer?**			0.665	0.415
Yes	677 (47.9)	736 (52.1)		
No	155 (45.5)	186 (54.5)		
**Does secondhand smoke cause adverse pregnancy outcomes?**			9.427	0.002
Yes	611 (49.8)	615 (50.2)		
No	221 (41.9)	307 (58.1)		

The husband’s willingness to quit smoking is closely related to the household atmosphere in which he resides. The study found that 59.6% of husbands in families with a total smoking ban expressed a desire to quit, compared to only 35.0% in families without smoking restrictions (p<0.05). Additionally, when family members held an anti-smoking attitude, the proportion of husbands wanting to quit smoking was 50.3%, significantly higher than the 32.1% observed in families where members were tolerant of smoking (p<0.05). Furthermore, the number of smokers present in the husband’s environment influences his willingness to quit: when there are fewer than two smokers nearby, the desire to quit smoking rises to 64.0% (p<0.05); however, this willingness decreases to 40.1% when more than three smokers are present (p<0.05) (Table 5). Detailed data are provided in Supplementary file Table 2.

**Table 5:** Univariate analysis of smoking cessation intention and household atmosphere, Shanghai, China, 2021–2024 (N=1754)

*Items*	*Willing to quit smoking n (%)*	*No desire to quit smoking n (%)*	*χ^2^*	*p*
**Is smoking allowed at home?**			13.945	<0.001
Yes	704 (53.3)	569 (44.7)		
No	218 (45.3)	263 (54.7)		
Family members’ attitude towards smoking*			30.557	<0.001
Permissive	88 (32.1)	186 (67.9)		
Opposed	744 (50.3)	736 (49.7)		
**Number of smokers around**			47.599	<0.001
0–1	174 (64.0)	98 (36.0)		
2–3	344 (49.2)	355 (50.8)		
4–5	314 (40.1)	469 (59.9)		

* Supportive: family members express explicit acceptance or tolerance toward the smoking behavior. Opposed: family members express explicit disapproval or actively encourage smoking cessation.

**Figure 1:**
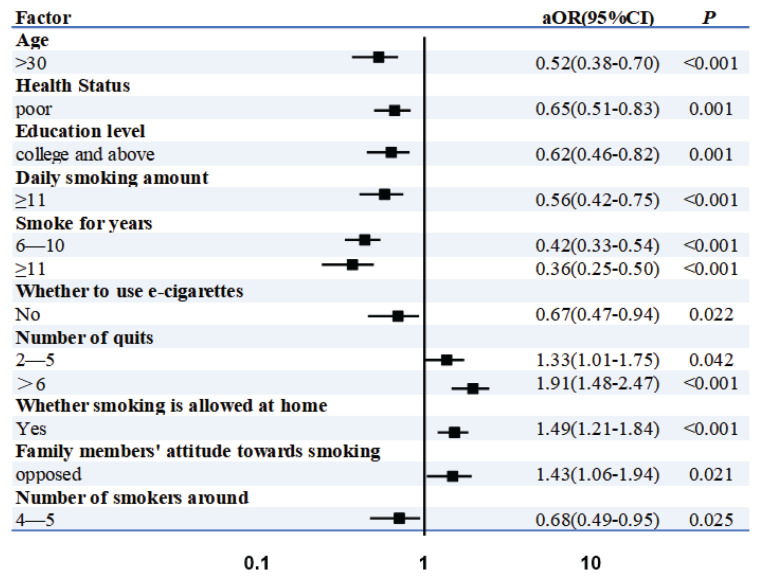
Logistic regression analysis results forest map

Logistic regression analysis indicated that, compared to husbands younger than 25 years old, those older than 35 years were less willing to quit smoking (AOR=0.52; 95% CI: 0.38–0.70, p<0.001). Additionally, husbands in poor health were less likely to quit smoking than their counterparts in good health (AOR=0.65; 95% CI: 0.50–0.93, p=0.001). Furthermore, husbands with a higher level of education were less likely to quit smoking compared to those with a lower education level (AOR=0.62; 95% CI: 0.46–0.82, p=0.001). When comparing smoking habits, husbands who smoked ≥11 cigarettes per day were less willing to quit than those who smoked ≤5 cigarettes daily (AOR=0.56; 95% CI: 0.42–0.75, p<0.001). Compared to husbands who smoked for ≤5 years, those with a smoking duration of ≥11 years were less likely to quit smoking (AOR=0.36; 95% CI: 0.25–0.50, p<0.001). Additionally, non-vaping husbands were less likely to quit smoking than their vaping counterparts (AOR=0.67; 95% CI: 0.47–0.94, p=0.022). In contrast, husbands who had never attempted to quit smoking more than five times were more likely to quit (AOR=1.91; 95% CI: 1.48-2.47, p<0.001). Furthermore, husbands from non-smoking families exhibited a higher likelihood of quitting smoking compared to those who permitted smoking in the home (AOR=1.49; 95% CI: 1.21–1.84, p < 0.001). Husbands whose family members opposed smoking demonstrated a greater intention to quit (AOR=1.43; 95% CI: 1.06–1.94, p=0.021) compared to those with fewer than two family members who smoked. Lastly, husbands who were surrounded by four to five smokers demonstrated significantly reduced odds of intending to quit smoking (AOR=0.68; 95% CI: 0.49–0.95, p=0.03) compared to those with fewer than two smokers in their immediate environment (reference group) (Figure 1).

## DISCUSSION

This study surveyed 1754 families of husbands who smoked during pregnancy in Shanghai. Multivariable analysis indicated that this intention was influenced by several factors, including the husband’s age, health status, and household environment. These findings underscore the interplay between individual characteristics and socio-environmental factors in shaping smoking cessation intentions during the perinatal period. The research findings demonstrate that a higher proportion of husbands in pregnant families expressed willingness to quit smoking compared to previously reported rates in studies conducted in China^15^ and South Korea^16^. This difference aligns with the focus on families during pregnancy, a critical physiological period in which heightened awareness of maternal and infant health may motivate expectant fathers to adopt positive health behaviors, including smoking cessation, to protect the well-being of both the mother and the child^17^.

The results of this study indicate that husbands in younger families exhibit a higher willingness to quit smoking compared to their counterparts in older families. This finding aligns with previous research that suggests younger populations tend to have more positive attitudes towards smoking cessation^18^. Specifically, younger husbands may demonstrate a greater enthusiasm for adopting healthier lifestyles, particularly during pregnancy. Concern for the health of their partners and future newborns may motivate them to take steps to quit smoking. Conversely, husbands with a higher level of education display a lower willingness to quit smoking compared to those with a level education. This conclusion contrasts significantly with relevant research findings from South Korea and the United States, where husbands with a lower level of education typically show a lower willingness to quit smoking. Such discrepancies may arise from variations in cultural backgrounds^19^. One possible explanation is that, while individuals with a higher level of education may possess substantial knowledge in their professional fields, this does not necessarily equate to higher health literacy or knowledge about smoking. In fact, a previous study revealed that among Chinese individuals with higher education, there was no statistically significant association between smoking cognition and smoking behavior among young male smokers^20,21^. As the number of pregnancies increases, husbands’ willingness to quit smoking tends to decrease. This trend may be associated with a ‘habitual’ mentality developed through multiple pregnancies, which diminishes the perceived necessity of quitting smoking^10^. Furthermore, smoking behavior among pregnant women negatively influences their husbands’ willingness to quit, suggesting a reciprocal relationship between smoking habits within the family. Specifically, when the pregnant woman is a smoker, her husband may be swayed by the family’s smoking culture, which in turn reduces his motivation to quit smoking^22^.

In a study investigating the association between husbands’ smoking habits and their willingness to quit, we identified several significant associational characteristics. Specifically, husbands who smoke larger daily amounts, have longer smoking histories, lack experience with e-cigarette use, have attempted to quit fewer times, and exhibit higher levels of nicotine dependence, demonstrate a relatively lower willingness to quit smoking. These findings align with those from a study conducted in Singapore^23^. It would be valuable to explore the implementation of differentiated health education measures and quit-smoking support services for husband groups with varying smoking habit characteristics in future research. In particular, husbands with severe nicotine dependence should receive increased attention and assistance. Additionally, a cautious approach should be adopted when assessing the auxiliary role of new tobacco products, such as e-cigarettes, in the quit-smoking process. Although some studies have reported positive effects of e-cigarettes on smoking cessation, their long-term effects and safety still require further scientific validation^24^.

The awareness level of husbands regarding non-lung health risks associated with smoking and secondhand smoke – such as cardiovascular diseases, erectile dysfunction, and adverse pregnancy outcomes – positively correlates with their willingness to quit smoking. According to the Health Belief Model, individuals with a greater intention to quit smoking are more likely to actively seek health-related information, thereby enhancing their knowledge base and deepening their understanding of the hazards of tobacco^25^. Additionally, the Theory of Planned Behavior underscores the importance of subjective norms, suggesting that the health concerns of spouses or family members may concurrently facilitate knowledge acquisition. By comprehending the health hazards of tobacco, husbands’ willingness to quit smoking can be further amplified^26^. However, this study is limited by its cross-sectional design, which precludes direct inference of causality. Future research should employ longitudinal designs or experimental methods to more accurately investigate the relationship between tobacco knowledge awareness and the willingness to quit smoking, thereby providing a theoretical framework for developing effective smoking cessation interventions.

Husbands’ willingness to quit smoking is greater when smoking is prohibited within the family, when wives express a strong opposition to smoking, and when there are fewer smokers in their vicinity. This suggests that a husband’s smoking cessation is closely linked to his immediate environment. When the surrounding population generally refrains from smoking and maintains a clear negative stance towards it, this social atmosphere creates an implicit pressure on the husband, effectively enhancing his determination and actions to quit smoking^22,27^. The results of this study highlight the potential importance of a comprehensive smoke-free environment as a factor associated with smoking cessation intentions. This finding underscores the theoretical and practical value of promotional, educational, and policy initiatives that target the broader social context of smoking. It suggests that future research should longitudinally examine whether concerted efforts across multiple sectors to shift public perceptions and attitudes can effectively nurture healthier social norms.

Logistic regression analysis has identified multiple factors influencing the willingness to quit smoking among husbands in pregnant and postpartum families. Specifically, age, health status, smoking behavior characteristics, and family environmental factors are significantly associated with this willingness. Husbands over the age of 35 years exhibit a markedly lower willingness to quit smoking compared to younger cohorts (OR=0.515), which may be linked to long-term nicotine dependence or a cognitive adaptation to the dangers of smoking^28^. Interestingly, husbands in poor health are paradoxically less inclined to quit smoking (OR=0.649). This may suggest that individuals with deteriorating health might increase their smoking behavior due to emotional regulation needs or difficulties associated with withdrawal, or it could indicate reverse causality (e.g. smoking worsening health issues)^29^. Additionally, the low willingness to quit among those with a higher level of education (OR=0.615) necessitates further reflection and investigation into the complex relationship between education level and smoking behavior. The intensity of smoking (≥11 cigarettes/day, OR=0.560) and the duration of smoking (≥11 years, OR=0.355) demonstrate a negative association with husbands’ willingness to quit smoking, indicating that the level of nicotine dependence is a barrier to smoking cessation^30^. The elevated quit intention among e-cigarette users may be attributed to their role as a cessation aid, which aligns with previous research findings that e-cigarettes can serve as substitutes for traditional cigarettes^31^. Additionally, the high quit intention observed in individuals who have attempted to quit smoking ≥5 times supports the self-efficacy theory, suggesting that prior behavioral experiences can bolster confidence in the ability to quit smoking^32^. The positive impact of household smoking bans and family members’ opposition to smoking underscores the importance of social support in facilitating behavioral change, consistent with the environmental reinforcement perspective of social cognitive theory^33^. Conversely, the negative association with a high number of surrounding smokers illustrates how peer influence may diminish motivation to quit, suggesting a potential detrimental effect of social circles on healthy behaviors^34^.

### Limitations

This study has several limitations that should be considered. First, the cross-sectional nature of our research design limits the ability to establish causal relationships between variables. A key methodological concern arising from this design is the potential for reverse causality, which is relevant to several of our observed associations. For instance, the relationship between poor health status and lower smoking cessation intention presents a classic challenge of directional ambiguity: it remains unclear whether poor health serves as a consequence of prolonged smoking or acts as a determinant that undermines cessation motivation. Second, the samples for this study were exclusively collected from the Jinshan District of Shanghai. Given the potential differences in socioeconomic conditions and cultural backgrounds between this region and other areas of China, the generalizability of the findings warrants cautious evaluation. Third, the data were primarily gathered through self-administered questionnaires, which may introduce recall bias or social desirability bias. For instance, some husbands may overestimate their willingness to quit smoking or underestimate their actual cigarette consumption.

## CONCLUSIONS

This study identified key factors influencing smoking cessation intention among husbands in pregnant families in Shanghai through a multidimensional analysis. The results demonstrated that cessation intention was significantly associated with individual characteristics (age, health status, education level), smoking behavior patterns (intensity, duration, nicotine dependence), and the household and social environment. Specifically, older age, poorer health status, higher nicotine dependence, and a more permissive household atmosphere were associated with lower quitting intention. These findings highlight the interplay of personal and environmental factors in shaping smoking cessation behaviors during the perinatal period. Future longitudinal studies are needed to clarify the temporal relationships between these variables and to support the development of more effective tobacco control strategies tailored to this population.

## Supplementary Material



## Data Availability

The data supporting this research are available from the authors on reasonable request.

## References

[1] Zhu S, Gao J, Zhang L, et al. Global, regional, and national cardiovascular disease burden attributable to smoking from 1990 to 2021: findings from the GBD 2021 Study. Tob Induc Dis. 2025;23(January):200072. doi:10.18332/tid/200072 PMC11784507 39897459

[2] Zhou M, Wang H, Zeng X, et al. Mortality, morbidity, and risk factors in China and its provinces, 1990–2017: a systematic analysis for the Global Burden of Disease Study 2017. Lancet. 2019;394:1145-1158. doi:10.1016/S0140-6736(19)30427-1 31248666 PMC6891889

[3] Flor LS, Anderson JA, Ahmad N, et al. Health effects associated with exposure to secondhand smoke: a burden of proof study. Nat Med. 2024;30:149-167. doi:10.1038/s41591-023-02743-4 38195750 PMC10803272

[4] Wang X, Zou W, Zhang X, et al. Exposure to secondhand smoke in indoor public places and attitudes of residents toward smoke control ordinances. Tob Induc Dis. 2024;22(December):196676. doi:10.18332/tid/196676 PMC11671798 39735278

[5] Pei D, Popova L, Chowdhury P, Shi J, Njie G. Exposure to anti- and pro-smoking messages among adults in China: results from the Global Adult Tobacco Survey, 2018. PLoS One. 2024;19(6):e0304028. doi:10.1371/journal.pone.0304028 38870150 PMC11175413

[6] Zhang L, Hsia J, Tu X, et al. Exposure to secondhand tobacco smoke and interventions among pregnant women in China: a systematic review. Prev Chronic Dis. 2015;12:E35. doi:10.5888/pcd12.140377 25789496 PMC4372160

[7] Marufu TC, Ahankari A, Coleman T, Lewis S. Maternal smoking and the risk of still birth: systematic review and meta-analysis. BMC Public Health. 2015;15:239. doi:10.1186/s12889-015-1552-5 25885887 PMC4372174

[8] Wang M, Wang ZP, Zhang M, Zhao ZT. Maternal passive smoking during pregnancy and neural tube defects in offspring: a meta-analysis. Arch Gynecol Obstet. 2014;289(3):513-521. doi:10.1007/s00404-013-2997-3 23942772

[9] Xia W, Li WHC, Cai W, et al. Smoking behavior among Chinese expectant fathers and smoking abstinence after partner pregnancy: a cross-sectional study. BMC Pregnancy Childbirth. 2020;20:449. doi:10.1186/s12884-020-03148-8 32758182 PMC7405418

[10] Yin H, Chen X, Zheng P, Kegler M, Shen Q, Xu B. A neglected opportunity for China’s tobacco control? Shift in smoking behavior during and after wives’ pregnancy. Tob Induc Dis. 2016;14(December):39. doi:10.1186/s12971-016-0105-8 27990102 PMC5148914

[11] Hemsing N, Greaves L, O’Leary R, Chan K, Okoli C. Partner support for smoking cessation during pregnancy: a systematic review. Nicotine Tob Res. 2012;14(7):767-776. doi:10.1093/ntr/ntr278 22180588

[12] Guindon GE, Driezen P, Chaloupka FJ, Fong GT. Cigarette tax avoidance and evasion: findings from the International Tobacco Control Policy Evaluation (ITC) Project. Tob Control. 2014;23 Suppl 1(0 1):i13-i22. doi:10.1136/tobaccocontrol-2013-051074 24227541 PMC4254713

[13] Huang CL, Lin HH, Wang HH. Psychometric properties of the Chinese version of the Fagerström Test for nicotine dependence. Addict Behav. 2006;31:2324-2327. doi:10.1016/j.addbeh.2006.02.024 16567055

[14] Berri KM, Adaba YK, Tarefasa TG, Bededa ND, Fekene DB. Maternal health service utilization from urban health extension professionals and associated factors among women who gave birth in the last one year in Ambo town, Oromia regional state, Ethiopia, 2018. BMC Public Health. 2020;20(1):499. doi:10.1186/s12889-020-08641-5 32295551 PMC7161097

[15] Huang ZX, Li YH, Xie Y, et al. Sociodemographic and smoking characteristics associated with intention to quit among Chinese adults: China Health Literacy Survey 2018–2019. Prev Med Rep. 2025;49:102933. doi:10.1016/j.pmedr.2024.102933 39691356 PMC11647633

[16] Jung M. Socio-contextual factors associated with male smokers’ intention to quit. BMC Public Health. 2016;16:398. doi:10.1186/s12889-016-3054-5 27178199 PMC4866397

[17] Cooper S, Orton S, Leonardi-Bee J, et al. Smoking and quit attempts during pregnancy and postpartum: a longitudinal UK cohort study. BMJ Open. 2017;7(11):e018746. doi:10.1136/bmjopen-2017-018746 PMC5695489 29146659

[18] Sun S, Yu H, Ling J, Yao D, Chen H, Liu G. The influence of health literacy and knowledge about smoking hazards on the intention to quit smoking and its intensity: an empirical study based on the data of China’s health literacy investigation. BMC Public Health. 2023;23(1):2355. doi:10.1186/s12889-023-17292-1 38017398 PMC10685583

[19] Gutiérrez-Torres DS, Reyes-Guzman C, Mayer M, Prutzman YM, Freedman ND. Quit attempts and use of cessation aids among U.S. adults who smoke nondaily. Am J Prev Med. 2025;68(3):622-626. doi:10.1016/j.amepre.2024.11.004 39571835 PMC11830521

[20] Xu X, Liu L, Sharma M, Zhao Y. Smoking-related knowledge, attitudes, behaviors, smoking cessation idea and education level among young adult male smokers in Chongqing, China. Int J Environ Res Public Health. 2015;12(2):2135-2149. doi:10.3390/ijerph120202135 25689992 PMC4344716

[21] Han HR, Kim J, Kim MT, Kim KB. Measuring health literacy among immigrants with a phonetic primary language: a case of Korean American women. J Immigr Minor Health. 2011;13(2):253-259. doi:10.1007/s10903-010-9366-0 20585985 PMC3010254

[22] Choi SH, Ling J, Noonan D, Kim W. Smoking behavior and social contexts associated with smoking among dual-smoker couples. Public Health Nurs. 2020;37(2):161-168. doi:10.1111/phn.12686 31724240

[23] Koh YS, Sambasivam R, AshaRani PV, et al. Factors influencing smoking cessation: evidence from Singapore’s nationwide health and lifestyle survey. Ann Acad Med Singap. 2024;53:608-620. doi:10.47102/annals-acadmedsg.2024177 39508693

[24] Gursoy E, Kaya R. Experiences, perceptions, and social dynamics of electronic cigarette users: a qualitative study. Health Expect. 2024;27:e70066. doi:10.1111/hex.70066 39400460 PMC11471881

[25] Kazemi A, Ehsanpour S, Nekoei-Zahraei NS. Promoting health beliefs to reduce environmental tobacco smoke exposure in pregnant women: a randomized trial. Health Educ Res. 2012;27:151-159. doi:10.1093/her/cyr102 22052216

[26] Tseng YF, Wang KL, Lin CY, Lin YT, Pan HC, Chang CJ. Predictors of smoking cessation in Taiwan: using the theory of planned behavior. Psychol Health Med. 2018;23(3):270-276. doi:10.1080/13548506.2017.1378820 28931309

[27] Fernando HN, Wimaladasa ITP, Sathkoralage AN, et al. Socioeconomic factors associated with tobacco smoking among adult males in Sri Lanka. BMC Public Health. 2019;19:778. doi:10.1186/s12889-019-7147-9 31215438 PMC6582511

[28] Fowler CD, Turner JR, Damaj MI. Molecular mechanisms associated with nicotine pharmacology and dependence. Handb Exp Pharmacol. 2020;258:373-393. doi:10.1007/164_2019_252 31267166

[29] Mackenbach JP, Damhuis RA, Been JV. The effects of smoking on health: growth of knowledge reveals even grimmer picture. Article in Dutch. Ned Tijdschr Geneeskd. 2017;160:D869. 28098043

[30] Guo Y, Liu DY, Wang YJ, et al. Family functioning and nicotine dependence among smoking fathers: a cross-sectional study. BMC Public Health. 2023;23:658. doi:10.1186/s12889-023-15475-4 37024859 PMC10080741

[31] Lin HX, Zhang Y, Chen MJ, et al. Patterns of e-cigarette use and association with cigarette cessation intention in China. Tob Induc Dis. 2022;20(February):16. doi:10.18332/tid/144251 35221859 PMC8832540

[32] Al Thani M, Leventakou V, Sofroniou A, et al. Factors associated with baseline smoking self-efficacy among male Qatari residents. PLoS One. 2022;17:e0263306. doi:10.1371/journal.pone.0263306 35085368 PMC8794180

[33] Latkin CA, Knowlton AR. Social network assessments and interventions for health behavior change: a critical review. Behav Med. 2015;41:90-97. doi:10.1080/08964289.2015.1034645 26332926 PMC4786366

[34] Orsal O, Ergun A. Effect of peer education on decision-making, self-efficacy, addiction, and smoking cessation among young people. Risk Manag Healthc Policy. 2021;14:925-945. doi:10.2147/RMHP.S280393 33716513 PMC7944371

