# Supplementary material for: The associated factors of smoking cessation intention among husbands in gestational households: A census-based cross-sectional survey
Source: Tob Induc Dis. 2026 Jan 9;24:10.18332/tid/213719. doi: 10.18332/tid/213719 (PMC12785702; doi:10.18332/tid/213719)
Supplement: Supplementary file 1 [file TID-24-01-s1.pdf]

Supplementary Table 1. Demographic characteristics of respondents (Shanghai, China), 2,021-2,024(N=1754)

Characteristics	Pregnant women N（%）	Husbands N（%）
**Age(year)**		
＜26	422（24.1）	117（6.7）
26-30	587（33.5）	535（30.5）
＞30	745（42.5）	1102（62.8）
**Household registration**		
Shanghai	432（24.6）	447（25.5）
Other places	1322（75.4）	1307（74.5）
**Education level**		
High school and below	960（54.7）	1431（81.6）
College and above	794（(45.3）	323（18.4）
**Occupation**		
peasantry	38（2.2）	73（4.2）
civil servant	70（4.0）	88（5.0）
private enterprises	508（29.0）	824（47.0）
professional technicians	117（6.7）	31（1.8）
Other positions	795（45.3）	717（40.9）
unemployed	226（12.9）	21（1.2）
**Number of pregnancies**		
1	700（39.9）	
2	511（29.1）	
≥3	543（31.0）	
**Total**	1754（100）	1754（100）

Supplementary Table 2. Logistic regression analysis of variable names and assignments

Variable	Assign
Husband's age（year）	＜26=1 26-30=2 ＞30=3
Husband's health	Good=1 Average=2 Poor=3
Chronic Illness Status	Yes=1 No=2
Education level	High school and below=1<br>College and above=2
Maternal age	＜26=1 26-30=2 ＞30=3
Number of pregnancies	1=1 2=2 3=3
Pregnant woman's smoking status	Active smoking=1<br>Passive smoking=2<br>No smoking=3
Daily smoking amount	≤5=1 6-10=2 ≥11=3
Smoke for years	≤5=1 6-10=2 ≥11=3
Whether to use e-cigarettes	Yes=1 No=2
Number of quits	0=1 1=2 2-5=3 ≥6=4
Nicotine dependence level	Mild=1 Moderate=2 Severe=3
Whether smoking causes stroke	Yes=1 No=2
Whether smoking causes heart disease	Yes=1 No=2
Whether smoking causes impotence	Yes=1 No=2
Whether secondhand smoke causes heart disease	Yes=1 No=2
Whether secondhand smoke causes adverse pregnancy	Yes=1 No=2
Whether smoking is allowed at home	Yes=1 No=2
Family members' attitude towards smoking	Permissive=1 Opposed=2
Number of smokers around	0-1=1 2-3=2 4-5=3

© 2026 Wang X. et al.
